# Life Cycle Assessment of Egg Production: Effects of Breed and Feeding a Low‐Protein Diet With Amino Acids During Laying Period

**DOI:** 10.1111/asj.70129

**Published:** 2025-11-11

**Authors:** Akira Setoguchi, Kazato Oishi, Akifumi Ogino, Hiroyuki Hirooka

**Affiliations:** ^1^ Laboratory of Animal Husbandry Resources, Division of Applied Biosciences, Graduate School of Agriculture Kyoto University Kyoto Japan; ^2^ Institute of Livestock and Grassland Science, National Agriculture and Food Research Organization (NARO) Ibaraki Japan

**Keywords:** egg production, environmental impact, layer production model, life cycle assessment, low‐protein diet

## Abstract

Feeding low‐protein diets supplemented with amino acids to poultry has been implemented to reduce the environmental impacts from excretion, while maintaining productivity. This study conducted a process‐based life cycle assessment of egg production to evaluate the effects of breed and feeding low‐protein diets (reduced by 2 percentage points in crude protein content) supplemented with amino acids (lysine, methionine, and tryptophan) to layers. The production system was simulated based on a nutrient requirement model and Japanese layer management guidelines. The system boundary was defined as the cradle‐to‐farm gate, and the environmental impacts were expressed per 1 kg of eggs produced. The environmental impact differences between white egg and brown egg layer breeds were also evaluated. As a result, the environmental assessment showed that feeding low‐protein diets reduced the impacts on global warming (by 5%), acidification (by 20%), eutrophication (by 14%), and energy consumption (by 2%), which were mainly caused by decreases in emissions generated from excretion. Compared with white egg layers, brown egg layers had larger environmental impacts due to their higher feed conversion ratio. This study quantitatively demonstrated how feeding a low‐protein diet supplemented with amino acids and differences in layer breeds affect environmental impacts.

## 1 Introduction

Interest in environmental issues has increased as the importance of Sustainable Development Goals (SDGs) has been recognized worldwide, and climate change becomes a major environmental issue. Gerber et al. (2013) reported that the livestock sector accounts for 14.5% of anthropogenic greenhouse gas (GHG) emissions. In poultry production, the main environmental pollutants are emitted from manure; in particular, nitrous oxide (N_2_O) has a high potential to cause greenhouse effects. Ammonia (NH_3_) emissions have been a topic of great concern as a cause of acidification and eutrophication (Bouwman et al. 2002) and as a source of indirect N_2_O emissions. Furthermore, NH_3_ emissions pose a risk to the health of both poultry and workers, making it necessary to implement pollutant mitigation strategies (Swelum et al. 2021; Bist et al. 2023).

Feeding a low‐protein diet is a promising technique that reduces crude protein (CP) content with supplementation of the limiting amino acids in the feed (Summers 1993; Blair et al. 1999). An appropriate control of CP and amino acids in feed could reduce the excreted nitrogen while maintaining productivity, leading to a decrease in N_2_O and NH_3_ emissions derived from excreted manure. Iio et al. (2021, 2023) reported that feeding a diet reduced by 2 percentage points in CP content and supplemented with amino acids to laying hens resulted in lower pollutant emissions from manure composting without affecting egg production performance.

Life cycle assessment (LCA) is a standard environmental assessment method that evaluates all related processes in a production system, including material exploitation, transport, and disposal (ISO 2006). Numerous studies have reported the environmental assessment of livestock production worldwide using LCA methodology (Cederberg and Mattsson 2000; Basset‐Mens and Van der Werf 2005; Mollenhorst et al. 2006). In terms of livestock production with low‐protein feeding, environmental assessments have been reported for swine (Ogino et al. 2013), broilers (Ogino et al. 2021), and dairy cattle (Setoguchi et al. 2022). There is a limited number of LCA studies evaluating the effects of feeding low‐protein diets to layers (Ershadi et al. 2021), although studies on low‐protein diets in egg production have been reported. To further investigate the effects of feeding a low‐protein diet on environmental impacts in detail, it is necessary to conduct an environmental assessment by a nutrient requirement model approach for layer production incorporating experimental data about the feed composition and the manure composting of layers fed a low‐protein diet. Thus, the objective of this study was to assess the effects of feeding a low‐protein diet with amino acids to layers on the environmental impacts of egg production systems designed based on previous studies. Moreover, this study analyzed the environmental impacts of two layer breeds, white and brown egg layers, which differ in growth and laying performance. According to a layer management guidebook (GHEN Corporation 2017, 2022), Julia, a white‐egg strain, is characterized by a smaller body weight and a higher peak laying rate, whereas Boris Brown, a brown‐egg strain, has an earlier onset of laying, larger egg weight, and higher survival rate. These breed‐specific traits were incorporated into the environmental assessment.

## 2 Materials and Methods

### 2.1 Description of the Layer Production System in the Present Study

To evaluate the environmental impacts of layer production in Japan, four systems were designed with combinations of two types of feed during the laying period (conventional diets [Conv] and low‐protein diets [LowCP]) and two representative layer breeds (Julia and Boris Brown: white egg and brown egg layers, respectively). Table 1 shows the feed composition during the laying periods. For the early and late stages of the laying period, two types of feed (Conv and LowCP) were formulated for each stage derived from the reports about feeding experiments by Iio et al. (2021, 2023). LowCP had 2 percentage points lower CP content compared to Conv and was adjusted by the supplementation of crystalline amino acids (lysine, methionine, and tryptophan). The feed composition during the rearing period was formulated based on published nutrient requirements (NARO 2011) (Table 2). The chemical composition was calculated based on the Standard Tables of Feed Composition in Japan (NARO 2010).

**TABLE 1:** Feed ingredients and chemical composition during laying period.

Items	To 51 weeks		After 51 weeks	
	Conv	LowCP	Conv	LowCP
Ingredient (% FM)				
Corn	57.5	61.6	64.8	69.1
Soybean meal	19.3	17.9	16.1	13.6
Corn gluten meal	9.4	6.5	3.0	1.0
Fish meal	—	—	2.5	2.5
Animal fat	1.0	1.0	1.0	1.0
Calcium carbonate	10.1	10.1	10.3	10.3
Dicalcium phosphate	1.7	1.7	1.3	1.3
Salt	0.4	0.4	0.3	0.3
Vitamin premix	0.2	0.2	0.2	0.2
Phytase	—	—	0.10	0.10
L‐lysine HCl	0.16	0.22	0.15	0.23
DL‐methionine	0.17	0.25	0.22	0.29
L‐tryptophan	0.01	0.03	0.06	0.07
Composition				
ME (kcal/kg)	2.8	2.8	2.8	2.8
CP (% FM)	20.2	18.0	16.0	14.0
Lysine^a^ (%)	0.93	0.91	0.88	0.86
Methionine^a^ (%)	0.51	0.55	0.50	0.53
Tryptophan^a^ (%)	0.19	0.19	0.22	0.21

Abbreviations: Conv, conventional diet; CP, crude protein; FM, fresh matter; LowCP, low‐protein diet; ME, metabolizable energy.

^a^ Values calculated based on NARO (2010).

**TABLE 2:** Feed ingredients and chemical composition during rearing period.

Items	To 4 weeks	4–10 weeks	10 weeks to laying
Ingredient (% FM)			
Corn	59.6	67.6	75.9
Sorghum	0.8	0.8	0.8
Rice	4.0	4.0	4.0
Corn gluten meal	1.8	1.8	1.8
DDGS	5.0	5.0	5.0
Soybean meal	21.8	14.0	6.0
Rapeseed meal	5.0	5.0	5.0
Calcium carbonate	1.8	1.6	1.3
Vitamin premix	0.2	0.2	0.2
Composition			
ME (kcal/kg)	2.9	3.0	3.1
CP (% FM)	19.0	16.1	13.2

Abbreviations: CP, crude protein; DDGS, distiller's dried grains with soluble; FM, fresh matter; ME, metabolizable energy.

In this study, the production system was simulated based on a nutrient requirement model of the layers (Hirooka 2020) (described in Appendix 0). The model consists of equations for calculating the growth, egg production and metabolizable energy for laying hens. The amounts of feed intake and egg production were calculated by the production system model. The parameters for growth and egg production for layers were simulated according to the guidebook (GHEN Corporation 2017, 2022); for example, the onset of laying was assumed to start at 147 and 143 days of age for white egg layers and brown egg layers, respectively. Since Iio et al. (2021, 2023) reported no significant difference in layer productivity between the two feeding types, it was assumed that there was no difference in the growth and egg production of layers due to the difference in diets in the present study. The chemical compositions in the excreted manure were calculated by the chemical compositions of the feed and the amount utilized by the laying hens.

### 2.2 Life Cycle Assessment

#### 2.2.1 Goal and Scope

**FIGURE 1:**
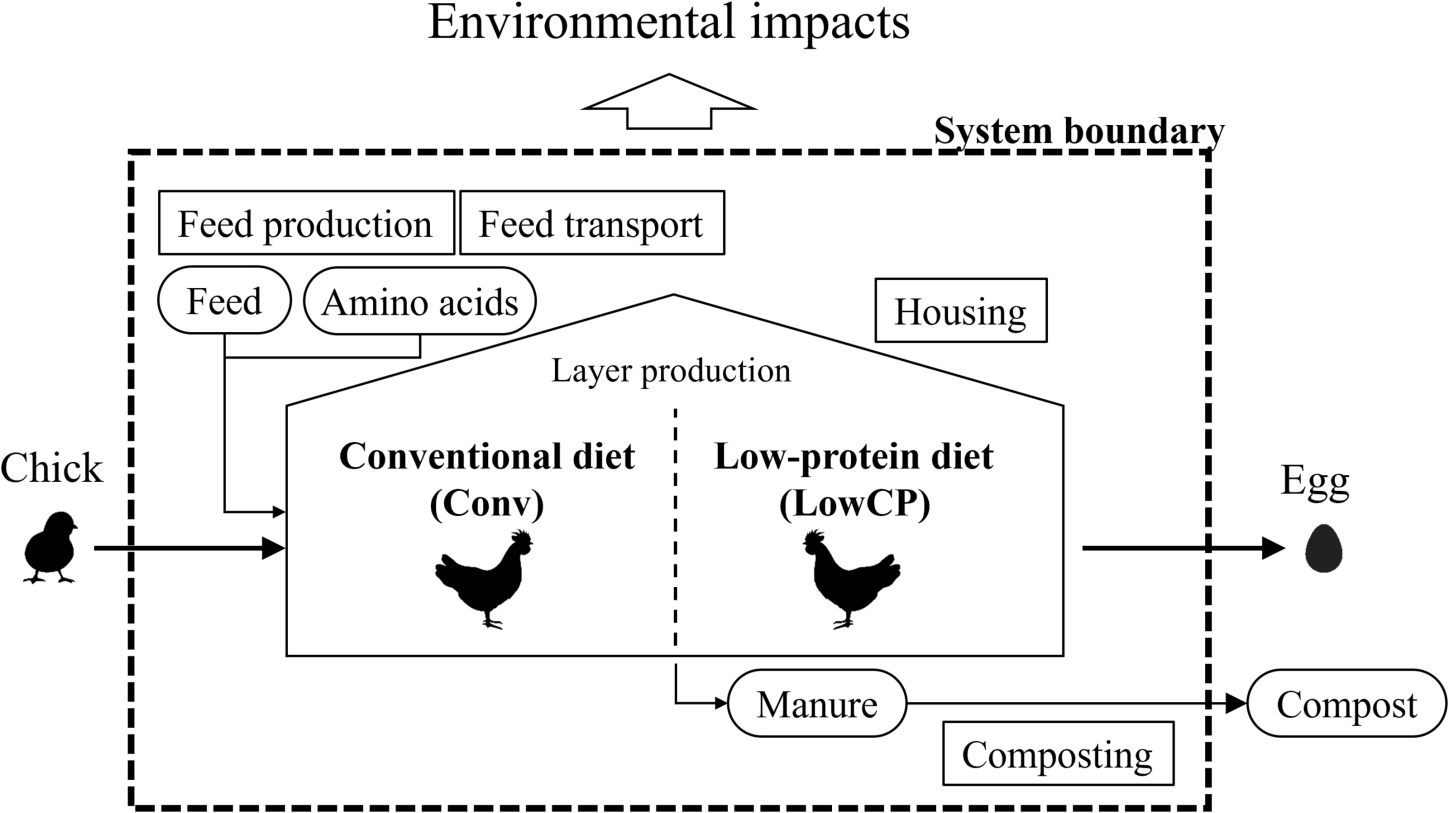
Outline of the layer production systems in this study.

The first step in LCA is to define the goal and scope of the analysis and the system boundaries. The goal of the analysis was to evaluate the environmental impacts of layer production systems that use low‐protein feeding. The system analyzed in this study is shown in Figure 1. The production system was modeled on a poultry farm in Japan using a cage system. The production processes included feed production, feed transport, animal housing, and composting. A functional unit was defined as 1 kg of eggs.

#### 2.2.2 Life Cycle Inventory

The second step in LCA is to draw an inventory of all resources and emissions related to all activities within the system boundaries. The emission factors associated with layer production considered in the analysis are shown in Table 3.

**TABLE 3:** Environmental loads associated with the layer production systems and output coefficients used in this study.

Source	Coefficient	Reference
Feed production and transport		
Energy use and relevant emissions	See the text	
Housing		
Energy use and relevant emissions	See the text	
NH_3_ (kg‐NH_3_‐N/kg‐N)	8.4%	MOE (2020)
Manure treatment		
CH_4_ (kg/kg‐OM)	Rearing period: 0.13% Early laying period: 0.15% (Conv), 0.07% (LowCP) Late laying period: 0.10% (Conv), 0.06% (LowCP)	MOE (2020) Iio et al. (2021) Iio et al. (2023)
N_2_O (kg‐N_2_O‐N/kg‐N)	Rearing period: 0.54% Early laying period: 0.48% (Conv), 0.53% (LowCP) Late laying period: 0.39% (Conv), 0.39% (LowCP)	MOE (2020) Iio et al. (2021) Iio et al. (2023)
NH_3_ (kg‐NH_3_‐N/kg‐N)	Rearing period: 51.5% Early laying period: 48.3% (Conv), 39.9% (LowCP) Late laying period: 59.3% (Conv), 49.5% (LowCP)	MOE (2020) Iio et al. (2021) Iio et al. (2023)
Indirect N_2_O emission		
N_2_O (kg‐N_2_O‐N/kg‐NH_3_‐N)	1.4%	IPCC (2019)

*Note:* Conv, conventional diet feeding; LowCP, low‐protein diet feeding; early laying period, from the start of egg production to 51 weeks of age; late laying period, 51 weeks of age or later.

Environmental substance emissions from feed production and transportation processes were calculated according to the methodology described by Ogino et al. (2021). Emissions from feed production were determined by the inputs of materials, energy, and relevant emissions. The emissions from the production of distiller's dried grains with solubles (DDGS) were calculated by assuming resource consumption of bioethanol (Canter et al. 2016) and reported economic allocation factors (Benavides et al. 2020). The emissions from crystalline amino acid production reported by Ogino et al. (2013) were used as the emissions from amino acid additives production. Emissions from the production of fat in feed were excluded, considering the low economic value of animal fat compared with meat. Emissions from feed transportation were calculated from the percentage of major exporting countries to Japan and transportation distances.

Poultry housing was assumed to be a cage system, which is at present a major system of egg production in Japan. The energy consumption of the facilities for layer housing and manure treatment was considered as the energy required for housing, and the environmental loads were calculated from the quantities of fuel and electricity used (AFFTIS 2000).

The emissions from the manure treatment were calculated by multiplying the amount of organic matter and nitrogen in the excreted manure by the emission factors. The manure treatment in the present study was assumed to be pile composting, and the values reported in the National Greenhouse Gas Inventory Report of Japan (MOE 2020) were used as emission factors before the laying period. In the laying period, the emission factors evaluated by Iio et al. (2021, 2023) were used.

Emissions of CO_2_ from respiration and excreted manure were not considered GHG emissions. The environmental loads associated with the production of capital goods, such as poultry houses and front loaders, were not considered.

#### 2.2.3 Life Cycle Impact Assessment

In this study, the contributions of the layer production systems were examined in relation to the environmental impact categories of global warming, acidification, eutrophication, and energy consumption. The contribution of the layer production system to global warming was expressed as CO_2_‐equivalent (CO_2_eq) factors, according to the Intergovernmental Panel on Climate Change (IPCC 2007): CO_2_, 1; CH_4_, 25; and N_2_O, 298, based on a time horizon of 100 years. To calculate the acidification potential of the different trace gases and the eutrophication potential of the different pollutants, SO_2_‐equivalent (SO_2_eq) factors for SO_X_, 1; NO_X_, 0.7; and NH_3_, 1.88, and PO_4_‐equivalent (PO_4_eq) factors for NO_X_, 0.13; NH_3_, 0.33; NO_3_, 0.1; and total phosphorus, 3.06 were used, which were derived from Heijungs et al. (1992).

### 2.3 Scenario Analysis

To examine the influence of variability in production parameters in the layer model developed in the present study, the following scenarios were evaluated by varying the parameter settings:

- BASE: baseline condition for white egg layers with Conv feeding
- FI: feed intake increased by 5% during the laying period without an increase in egg production
- EGG: laying rate decreased by 5% without a reduction in feed intake
- SUR: survival rate at the onset of laying unchanged, but decreased by 5% at the end of laying.

## 3 Results and Discussion

### 3.1 Simulated Results of the Layer Production Systems

**FIGURE 2:**
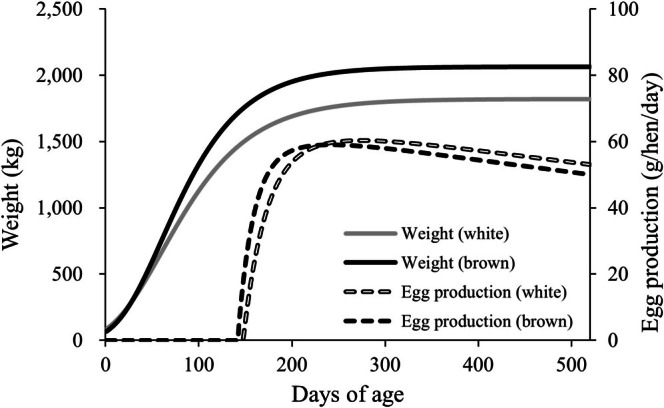
Body weight and egg production of white egg and brown egg layers.

The daily growth, egg production, and nutrient excretion of each layer breed were simulated using a layer production model (Figure 2). Table 4 presents the simulated results of the layer production systems in this study. For the white egg layers with Conv feeding, feed consumption and egg production until 520 days of age were 43.6 and 20.7 kg, respectively. LowCP feeding reduced nitrogen intake by 12% and nitrogen excretion by 20%. Comparing white egg layers with Conv feeding, brown egg layers increased feed intake despite a small increase in egg production. The white egg layers had a feed conversion ratio of 2.15, whereas the brown egg layers had a feed conversion ratio of 2.31.

**TABLE 4:** Simulated results for the layer production systems using a nutrient requirement model of layers.

Items	White		Brown	
	Conv	LowCP	Conv	LowCP
Final body weight (g)	1819	1819	2065	2065
Egg production (kg)	20.7	20.7	20.2	20.2
Feed consumption (kg)	43.6	43.4	46.7	46.6
Nitrogen intake (N‐kg)	1.21	1.09	1.30	1.17
Nitrogen excretion (N‐kg)	0.77	0.64	0.85	0.71

Abbreviations: Conv, conventional diet feeding; LowCP, low‐protein diet feeding.

Iio et al. (2021) reported that, compared with feeding a control diet (CP content of 19%), feeding a low‐protein diet with amino acids (CP content of 17%) to laying hens from 200 to 300 days of age reduced daily nitrogen excretion by 18.9% without decreasing egg production. Furthermore, Iio et al. (2023) reported that feeding a low‐protein diet with amino acids (CP content of 14%) to laying hens from 330 to 545 days of age reduced nitrogen excretion by 21.0% compared with a control diet feeding (CP content of 16%) without negative effects on egg production. Considering that nitrogen excretion included both the rearing and laying periods in the present study, the reduction in total nitrogen excretion (which ranged from 16.5% to 16.9%) by a low‐protein diet feeding evaluated using the model falls within a reasonable range. Nitrogen excretions of white egg layers with conventional and low‐protein diets estimated by the model in the present study were 2.07 and 1.73 g/day in the early laying period, and 1.55 and 1.22 g/day in the late laying period, respectively. On average, these values were 0.16 g/day higher than those reported in Iio et al. (2021, 2023). Although the discrepancy was small, it could be explained by differences in hen‐day egg mass. Iio et al. (2021, 2023) reported higher egg mass than that assumed in the present study. As a result, a smaller proportion of dietary nitrogen was allocated to egg production in the present study, resulting in a higher amount of nitrogen calculated as manure excretion.

The present model was developed under the assumption that LowCP diet does not affect layer productivity. Nevertheless, the effect of low‐protein feeding on productivity has been extensively investigated. Liang et al. (2005), by measuring NH_3_ emissions in hen houses, reported that a percentage point reduction in the CP content of feed led to a 10% reduction in NH_3_ without affecting hen‐day egg production. Ji et al. (2014) reported that there were no differences in egg production between feeds with 18% and 17% CP content in laying hens aged 21 to 34 weeks whereas feeds containing 16.5% and 16.0% CP negatively affected productivity. Therefore, these studies suggest that productivity can be maintained with a moderate reduction in feed CP content.

### 3.2 Environmental Impacts of Egg Production

**FIGURE 3:**
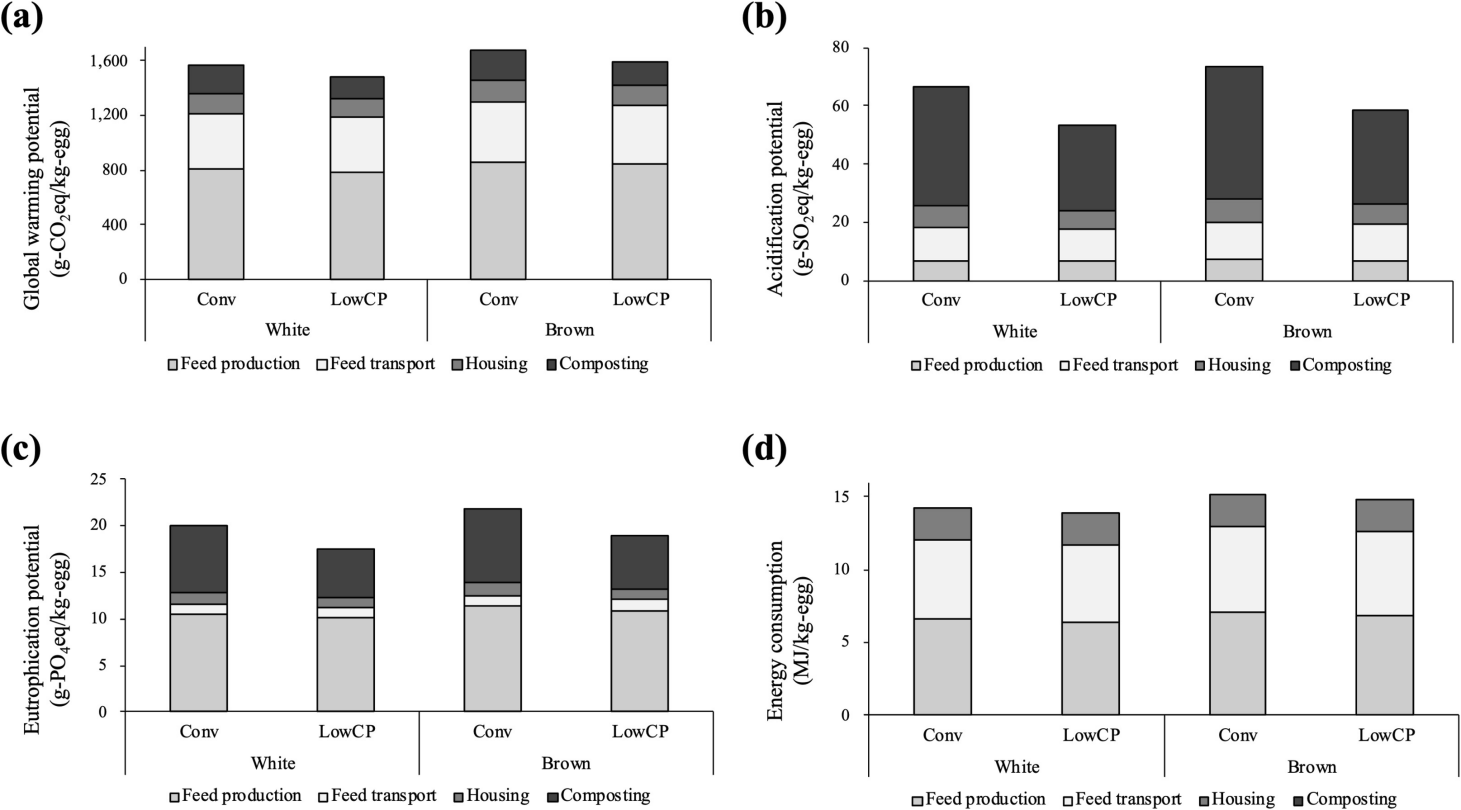
Environmental impacts of four‐layer production systems. Global warming potential (a), acidification potential (b), eutrophication potential (c), and energy consumption (d). Conv: conventional feeding, LowCP: low‐protein feeding.

**FIGURE 4:**
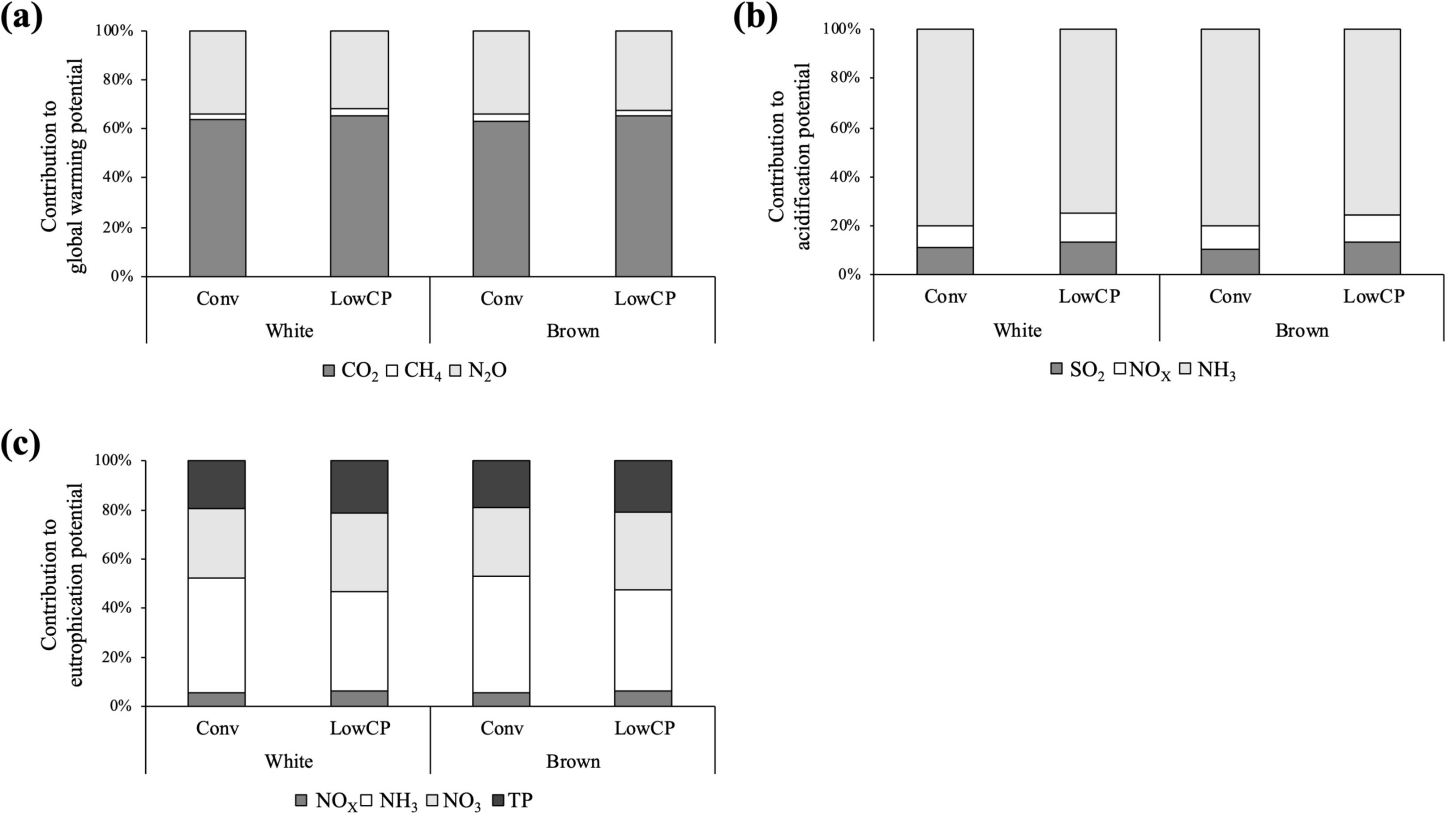
Contribution of substances to environmental impacts. Global warming potential (a), acidification potential (b), and eutrophication potential (c). Conv: conventional feeding, LowCP: low‐protein feeding.

Figures 3 and 4 show the results of environmental assessment in the present study. For white egg layers with Conv feeding, environmental impacts on global warming, acidification, eutrophication, and energy consumption per 1 kg of eggs were 1560 g‐CO_2_eq/kg‐egg, 66.9 g‐SO_2_eq/kg‐egg, 20.1 g‐PO_4_eq/kg‐egg, and 14.2 MJ/kg‐egg, respectively. Regarding global warming and energy consumption, the impacts related to feed accounted for a large share being about 80%. On the other hand, composting accounted for a large share of impacts on acidification and eutrophication, mostly due to NH_3_ emission. Using LowCP diet reduced the impact on global warming, acidification, eutrophication, and energy consumption by 5%, 20%, 14%, and 2%, respectively. In the case of Conv feeding, brown egg layers showed higher environmental impacts on global warming, acidification, eutrophication, and energy consumption than brown egg layers by 7%, 9%, 8%, and 6%, respectively.

In comparison with previous studies, Copley et al. (2023) calculated the global warming potential to be 1200 g‐CO_2_/kg‐egg for the cage system of Australian egg production, which is slightly lower than the results of the present study. Leinonen et al. (2012) assessed the environmental impacts of egg production systems in the United Kingdom and reported that the environmental impacts of the layer production with a cage system were 2920 g‐CO_2_eq/kg‐egg, 53.1 g‐SO_2_eq/kg‐egg, 18.5 g‐PO_4_eq/kg‐egg, and 16.9 MJ/kg‐egg on global warming, acidification, eutrophication, and energy consumption, respectively. Pelletier (2017) assessed the environmental impacts of Canadian egg production systems and reported 2443 g‐CO_2_eq/kg‐egg, 78.4 g‐SO_2_eq/kg‐egg, 24.4 g‐PO_4_eq/kg‐egg, and 11.2 MJ/kg‐egg on global warming, acidification, eutrophication, and energy consumption, respectively, as a conventional cage system. These studies showed a much larger impact on global warming than that of the present study. One of the major reasons was that the emissions of feed production per unit were quite different. Leinonen et al. (2012) included GHG emissions from land‐use change in their system boundary, which has significant effects on the result of the environmental assessment. On the other hand, Pelletier (2017) evaluated large emissions of animal fats used for feed (58.2 t‐CO_2_eq/t for tallow compared to 0.33 t‐CO_2_eq/t for corn). In the present study, emissions of animal fat for feed were not included due to its low economic value. Since the environmental loads of byproducts significantly depend on the allocation method (Cherubini et al. 2018), it is necessary to note the treatment of byproducts when comparing the results of LCA studies. Pelletier (2017) also included packaging in the system boundary, which is another reason for the larger emissions.

### 3.3 Effect of Low‐Protein Feeding

There have been a limited number of studies to assess the environmental impacts of low‐protein feeding supplemented with amino acids. Ogino et al. (2013) evaluated environmental impacts from pig production, resulting in a low‐protein feeding reducing global warming potential by 5.4% and the eutrophication potential by 28%. In broiler production, Ogino et al. (2021) showed that a low‐protein feeding reduced environmental impacts on global warming, acidification, and eutrophication by 2%, 19%, and 10%, respectively. Results of the present study showed that environmental impacts were similarly reduced by low‐protein feeding also in egg production.

The environmental impacts affected by feeding low‐protein diets are mainly related to feed production and manure nitrogen. The quantity of synthetic amino acids incorporated into feed formulations was minimal, and consequently the environmental impacts associated with their production have been reported to be relatively small within the overall feed production (Mosnier et al. 2011; Ogino et al. 2013; Ogino et al. 2021). On the other hand, Ershadi et al. (2021) reported that feeding a low‐protein diet supplemented with amino acids largely increased energy consumption per 1 kg of feed. This increase was attributed to the relatively high supplementation levels of synthetic amino acids in the diet (about 1.8%), which contributed to greater energy consumption of overall feed production. In terms of manure nitrogen, feeding a low‐protein diet reduced the emissions from manure management. In the present study, the low‐protein feeding reduced the potential for acidification and eutrophication by decreasing the emission of NH_3_ as well as the amount of excreted nitrogen. Iio et al. (2023) indicated that low‐protein feeding increased the C:N ratio in the manure which could influence NH_3_ emission. Thus, the use of low‐protein diets has the potential to substantially mitigate emissions of nitrogen‐related environmental impacts. Further investigations are required to advance its practical implementation.

### 3.4 Effect of Layer Breeds

The present study showed that the environmental impact per unit of egg weight was lower for white egg layers than for brown egg layers. However, this result is condition specific, as changes in housing systems or management conditions can alter the relative efficiency of different breeds. For example, Ghaly and El‐Husseiny (2021) reported that the profitability of brown egg layers was higher than that of white egg layers in non‐cage systems in Egypt, since brown egg layers were designed to achieve both a high egg weight and a high egg‐laying rate in such systems. Therefore, brown egg layers may have higher productivity and lower environmental impacts than white egg layers under these production conditions. In addition, when economic aspects such as consumer preferences and price premiums for brown eggs are taken into account, brown egg layers may also perform better than white egg layers in terms of environmental impacts per unit of economic value. Rondoni et al. (2020) reviewed studies on consumer egg preferences and noted that brown eggs were preferred over white eggs in several countries. For instance, Chen et al. (2023) showed that Chinese consumers tended to prefer colored eggs to white eggs. Chang et al. (2010) investigated the factors affecting the retail price of eggs in the United States using hedonic analysis and showed a price premium for brown eggs. Thus, considering economic aspects, the relative advantage of white or brown egg layers may vary depending on production systems and market conditions. Further environmental impact assessments that incorporate economic aspects will be required.

### 3.5 Scenario Analysis

The results of the scenario analysis are presented in Table 5. Increasing feed intake by 5% resulted in increases of 4.4%, 6.4%, 5.5%, and 3.8% in global warming potential, acidification potential, eutrophication potential, and energy use, respectively. When laying rate was decreased by 5%, the corresponding increases were 5.6%, 7.2%, 6.4%, and 5.3%, respectively. A decrease of 5% survival rate at the end of laying caused only slight increases in environmental impacts. These results indicate that variations in laying rate, which directly affect egg output as the functional unit in LCA, had the largest influence on environmental impacts. Costantini et al. (2020), evaluating the environmental impacts of organic egg production in Northern Italy, reported through sensitivity analysis that reducing the hen‐day egg production rate from 90% to 85% increased the global warming potential by 6.1%. Similarly, Weeks et al. (2016), in a meta‐analysis based on 10 studies, found that egg yield was the most influential factor determining environmental impacts, whereas the contribution of cumulative mortality was relatively minor. The results of the present study showed a similar pattern, confirming that parameters related to egg production have a dominant influence on environmental impacts. Overall, the present study highlights the importance of egg production traits, such as laying rate, in determining the environmental impacts and also provides a foundation for more comprehensive evaluations of the Japanese egg industry.

**TABLE 5:** Scenario analysis of environmental impacts under changes in feed intake, laying rate, and survival rate.

	BASE	FI	EGG	SUR
GWP (g‐CO_2_eq/kg‐egg)	1534	1601	1620	1543
AP (g‐SO_2_eq/kg‐egg)	66.5	70.8	71.3	66.8
EP (g‐PO_4_eq/kg‐egg)	19.8	20.9	21.0	19.9
EC (MJ/kg‐egg)	13.9	14.4	14.6	14.0

*Note:* BASE: baseline condition of white egg layer with a conventional diet, FI: 5% increase in feed intake during the laying period, EGG: 5% decrease in laying rate, SUR: 5% decrease in survival rate at the end of laying, GWP: global warming potential, AP: acidification potential, EP: eutrophication potential, EC: energy consumption.

## 4 Conclusions

In the present study, an environmental assessment was conducted to evaluate the environmental impacts of egg production by feeding low‐protein diets supplemented with amino acids in Japan using a nutrient requirement model for layer production systems. The results showed that a 2 percentage points reduction in feed protein content during the laying period reduced environmental impacts in all categories evaluated. In particular, the impacts on acidification and eutrophication were reduced (by 20% and 13%), mainly due to reduced nitrogen excretion and the resulting N_2_O and NH_3_ emissions. In addition, the results indicated that brown egg layers had higher environmental impacts than white egg layers due to their higher feed conversion ratio in the cage system. As the present study did not account for differences in management systems (cage system or cage‐free and organic systems) or consumers' economic valuation of eggs between the two types of layers, further analyses investigating the economic and environmental performance of brown egg layers will be required.

## Conflicts of Interest

The authors declare no conflicts of interest.

## Description of nutrient requirement model of layer

In this study, feed requirements, egg production, and nutrient excretion of the layers were calculated using mathematical models. The selection of the formula was based on a study by Hirooka (2020), and the formula parameters related to live weight and egg laying rate were updated using published data from layer management guides (GHEN Corporation 2017, 2022). In addition, the change in egg weight with age was considered based on layer management guidelines.

The live weight of layers at t days of age ($W_{t}$; g) and egg weight at $t_{p}$ days from the start of egg production (${\mathrm{EW}}_{t_{p}}$; g) were assumed to follow the Gompertz curve, and the egg laying rate (${\mathrm{ER}}_{t_{p}}$; %) was fitted to the equation reported by McMillan (1981). The fitted equations estimated using the nls package in R software (ver. 4.3.1) are as follows:

For white egg layers:

(1)
$$W_{t}=1817.2\exp\left(-3.09\exp\left(-0.019t\right)\right)$$

(2)
$${\mathrm{EW}}_{t_{p}}=65.60\exp\left(-3.43\exp\left(-0.017t_{p}\right)\right)$$

(3)
$${\mathrm{ER}}_{t_{p}}=103.8\left(1-\exp\left(-0.0480\left(t_{p}-0.492\right)\right)\right)\times \exp\left(-0.00052t_{p}\right)$$

For brown egg layers:

(4)
$$W_{t}=2065.1\exp\left(-3.48\exp\left(-0.021t\right)\right)$$

(5)
$${\mathrm{EW}}_{t_{p}}=64.94\exp\left(-3.82\exp\left(-0.020t_{p}\right)\right)$$

(6)
$${\mathrm{ER}}_{t_{p}}=101.0\left(1-\exp\left(-0.0673\left(t_{p}+1.365\right)\right)\right)\times \exp\left(-0.00059t_{p}\right)$$

It was assumed that the survival rate changed linearly from the start of egg laying until 80 weeks of age. At 80 weeks of age, the survival rates were 94% and 95% for white and brown egg layers, respectively. Hirooka (2020) calculated the amount of fat and protein accumulation in layers ($F_{t}$; g and $P_{t}$; g, respectively) and the intake of metabolizable energy (${\mathrm{ME}}_{t}$; kcal/d) by applying reported data from broilers and layers (Sakomura et al. 2003; Sakomura 2004) due to the lack of information on layers. In the present study, ${\mathrm{ME}}_{t}$ during the rearing period was calculated using the following equation reported by Hirooka (2020):

(7)
$$F_{t}=0.16890{W_{t}}^{0.907}$$

(8)
$$P_{t}=0.17345{W_{t}}^{1.0233}$$

(9)
$${ME}_{t}=92.40{W_{t}}^{0.75}+13.52∆F_{t}+12.59∆P_{t}$$

where $∆F_{t}$ and $∆P_{t}$ in Equation (9) are the daily accumulations of fat and protein, respectively, which were obtained by differentiating Equations (7) and (8). ${\mathrm{ME}}_{t}$ during the laying period was evaluated using the following equation reported by the Japanese Feeding Standard for Poultry (NARO 2011):

(10)
$${\mathrm{ME}}_{t}=110{W_{t}}^{0.75}\frac{-0.081{\left(22-T\right)}^{2}+2\left(22-T\right)+94}{94}+2.2{\mathrm{ER}}_{t_{p}}{\mathrm{EW}}_{t_{p}}+2{\mathrm{DG}}_{t}$$

where T is the temperature in the layer house, which was set to 22°C in the present study, and ${\mathrm{DG}}_{t}$; g is the daily gain at t days. Feed intake of the layers was calculated to meet the ME requirements.

The nitrogen content of the manure was calculated by subtracting the protein retained for growth and eggs from the CP intake. The CP content of the eggs was 12% (NARO 2011), and the nitrogen‐to‐protein conversion factor was 6.25. The organic matter in the manure was calculated by subtracting the crude ash and digestible nutrient contents from the dry matter content in the diets.

## References

[asj70129-bib-0001] Agriculture, Forestry and Fisheries Technology Information Society (AFFTIS) . 2000. “Investigation of Energy‐Managing Agricultural Production System Development (in Japanese).” Tokyo: AFFTIS.

[asj70129-bib-0002] Basset‐Mens, C. , and H. M. Van der Werf . 2005. “Scenario‐Based Environmental Assessment of Farming Systems: The Case of Pig Production in France.” Agriculture, Ecosystems & Environment 105: 127–144. 10.1016/j.agee.2004.05.007.

[asj70129-bib-0003] Benavides, P. T. , H. Cai , M. Wang , and N. Bajjalieh . 2020. “Life‐Cycle Analysis of Soybean Meal, Distiller‐Dried Grains With Solubles, and Synthetic Amino Acid‐Based Animal Feeds for Swine and Poultry Production.” Animal Feed Science and Technology 268: 114607. 10.1016/j.anifeedsci.2020.114607.

[asj70129-bib-0004] Bist, R. B. , S. Subedi , L. Chai , and X. Yang . 2023. “Ammonia Emissions, Impacts, and Mitigation Strategies for Poultry Production: A Critical Review.” Journal of Environmental Management 328: 116919. 10.1016/j.jenvman.2022.116919. 36516703

[asj70129-bib-0005] Blair, R. , J. P. Jacob , S. Ibrahim , and P. Wang . 1999. “A Quantitative Assessment of Reduced Protein Diets and Supplements to Improve Nitrogen Utilization.” Journal of Applied Poultry Research 8: 25–47. 10.1093/japr/8.1.25.

[asj70129-bib-0006] Bouwman, A. F. , D. P. Van Vuuren , R. G. Derwent , and M. Posch . 2002. “A Global Analysis of Acidification and Eutrophication of Terrestrial Ecosystems.” Water, Air, and Soil Pollution 141: 349–382. 10.1023/A:1021398008726.

[asj70129-bib-0007] Canter, C. E. , J. B. Dunn , J. Han , Z. Wang , and M. Wang . 2016. “Policy Implications of Allocation Methods in the Life Cycle Analysis of Integrated Corn and Corn Stover Ethanol Production.” Bioenergy Research 9: 77–87. 10.1007/s12155-015-9664-4.

[asj70129-bib-0008] Cederberg, C. , and B. Mattsson . 2000. “Life Cycle Assessment of Milk Production—a Comparison of Conventional and Organic Farming.” Journal of Cleaner Production 8: 49–60. 10.1016/S0959-6526(99)00311-X.

[asj70129-bib-0009] Chang, J. B. , J. L. Lusk , and F. B. Norwood . 2010. “The Price of Happy Hens: A Hedonic Analysis of Retail Egg Prices.” Journal of Agricultural and Resource Economics 35: 406–423.

[asj70129-bib-0010] Chen, R. , C. Jiang , X. Li , et al. 2023. “Research on Chinese Consumers' Shell Egg Consumption Preferences and the Egg Quality of Functional Eggs.” Poultry Science 102: 103007. 10.1016/j.psj.2023.103007. PMC10462883 37598555

[asj70129-bib-0011] Cherubini, E. , D. Franco , G. M. Zanghelini , and S. R. Soares . 2018. “Uncertainty in LCA Case Study due to Allocation Approaches and Life Cycle Impact Assessment Methods.” International Journal of Life Cycle Assessment 23: 2055–2070. 10.1007/s11367-017-1432-6.

[asj70129-bib-0012] Copley, M. A. , S. G. Wiedemann , and E. J. McGahan . 2023. “Environmental Impacts of the Australian Poultry Industry. 2. Egg Production.” Animal Production Science 63: 505–521. 10.1071/AN22297.

[asj70129-bib-0013] Costantini, M. , D. Lovarelli , L. Orsi , et al. 2020. “Investigating on the Environmental Sustainability of Animal Products: The Case of Organic Eggs.” Journal of Cleaner Production 274: 123046. 10.1016/j.jclepro.2020.123046.

[asj70129-bib-0014] Ershadi, S. Z. , M. D. Heidari , B. Dutta , G. Dias , and N. Pelletier . 2021. “Comparative Life Cycle Assessment of Technologies and Strategies to Improve Nitrogen Use Efficiency in Egg Supply Chains.” Resources, Conservation and Recycling 166: 105275. 10.1016/j.resconrec.2020.105275.

[asj70129-bib-0015] Gerber, P. J. , H. Steinfeld , B. Henderson , et al. 2013. “Tackling Climate Change Through Livestock: A Global Assessment of Emissions and Mitigation Opportunities. Rome: Food and Agriculture Organization of the United Nations (FAO).” Accessed March 5, 2025. https://www.fao.org/3/i3437e/i3437e00.htm.

[asj70129-bib-0016] Ghaly, M. M. , and O. El‐Husseiny . 2021. “Best‐Fitted Regression Models for Profitability of Two Egg‐Type Commercial Pullets.” Tropical Animal Health and Production 53: 204. 10.1007/s11250-021-02580-y. 33710433

[asj70129-bib-0017] GHEN Corporation . 2017. “Layer Management Guide, Boris Brown (in Japanese).” Gifu: GHEN Corporation. Accessed March 5, 2025. https://www.ghen.co.jp/pdf/04‐borisbrown.pdf.

[asj70129-bib-0018] GHEN Corporation . 2022. “Layer Management Guide, Julia (In Japanese). GHEN Corporation, Gifu.” Accessed March 5, 2025. https://www.ghen.co.jp/pdf/01‐julia.pdf.

[asj70129-bib-0019] Heijungs, R. , J. B. Guinée , G. Huppes , et al. 1992. “Environmental Life Cycle Assessment of Products: Guide. Leiden, the Netherlands: Center of Environmental Science (CML).” Leiden University.

[asj70129-bib-0020] Hirooka, H. 2020. “Development of Simulation Models for Life Cycle Layer and Broiler Production and Nitrogen Utilization (In Japanese).” Nihon Chikusan Gakkaiho 91: 17–22. 10.2508/chikusan.91.17.

[asj70129-bib-0021] Iio, W. , R. Shimada , I. Nonaka , and A. Ogino . 2023. “Effects of a Low‐Protein Diet Supplemented With Essential Amino Acids on Egg Production Performance and Environmental Gas Emissions From Layer‐Manure Composting in Laying Hens in the Later Laying Period.” Animal Science Journal 94: e13853. 10.1111/asj.13853. 37431230

[asj70129-bib-0022] Iio, W. , K. Yamashita , R. Shimada , A. Ogino , I. Nonaka , and T. Osada . 2021. “Effects of a Lower Crude Protein Diet on Egg Production in 200‐300‐Day‐Old Layers and Environmental Load Gas Emission During Composting of Their Manure (In Japanese).” Nihon Chikusan Gakkaiho 92: 485–491. 10.2508/chikusan.92.485.

[asj70129-bib-0023] Intergovernmental Panel on Climate Change (IPCC) . 2007. “Climate Change 2007: The Physical Science Basis.” Accessed March 5, 2025. https://www.ipcc.ch/report/ar4/wg1/.

[asj70129-bib-0024] Intergovernmental Panel on Climate Change (IPCC) . 2019. “2019 Refinement to the 2006 IPCC Guidelines for National Greenhouse Gas Inventories.” Accessed March 5, 2025. https://www.ipcc‐nggip.iges.or.jp/public/2019rf/vol4.html.

[asj70129-bib-0025] International Organization for Standardization (ISO) . 2006. “Environmental Management – Life Cycle Assessment: Principles and Framework.” Switzerland: ISO.

[asj70129-bib-0026] Ji, F. , S. Y. Fu , B. Ren , et al. 2014. “Evaluation of Amino‐Acid Supplemented Diets Varying in Protein Levels for Laying Hens.” Journal of Applied Poultry Research 23: 384–392. 10.3382/japr.2013-00831.

[asj70129-bib-0027] Leinonen, I. , A. G. Williams , J. Wiseman , J. Guy , and I. Kyriazakis . 2012. “Predicting the Environmental Impacts of Chicken Systems in the United Kingdom Through a Life Cycle Assessment: Egg Production Systems.” Poultry Science 91: 26–40. 10.3382/ps.2011-01635. 22184425

[asj70129-bib-0028] Liang, Y. , H. Xin , E. F. Wheeler , et al. 2005. “Ammonia Emissions From US Laying Hen Houses in Iowa and Pennsylvania.” Transactions of ASAE 48: 1927–1941. 10.13031/2013.20002.

[asj70129-bib-0029] McMillan, I. A. N. 1981. “Compartmental Model Analysis of Poultry Egg Production Curves.” Poultry Science 60: 1549–1551. 10.3382/ps.0601549.

[asj70129-bib-0030] Ministry of the Environment, Government of Japan (MOE) . 2020. “National Greenhouse Gas Inventory Report of Japan.” Accessed March 5, 2025. https://www.nies.go.jp/gio/archive/nir/jqjm1000000pcibe‐att/NIR‐JPN‐2020‐v3.0_GIOweb.pdf.

[asj70129-bib-0031] Mollenhorst, H. , P. B. M. Berentsen , and I. J. M. De Boer . 2006. “On‐Farm Quantification of Sustainability Indicators: An Application to Egg Production Systems.” British Poultry Science 47: 405–417. 10.1080/00071660600829282 16905466

[asj70129-bib-0032] Mosnier, E. , H. M. G. Van der Werf , J. Boissy , and J. Y. Dourmad . 2011. “Evaluation of the Environmental Implications of the Incorporation of Feed‐Use Amino Acids in the Manufacturing of Pig and Broiler Feeds Using Life Cycle Assessment.” Animal 5: 1972–1983. 10.1017/S1751731111001078. 22440474

[asj70129-bib-0033] National Agriculture and Food Research Organization (NARO) . 2010. “Standard Tables of Feed Composition in Japan, 2009 (in Japanese).” Tokyo: Japan Livestock Industry Association.

[asj70129-bib-0034] National Agriculture and Food Research Organization (NARO) . 2011. “Japanese Feeding Standard for Poultry (in Japanese). Tokyo: Japan Livestock Industry Association”.

[asj70129-bib-0035] Ogino, A. , K. Oishi , A. Setoguchi , and T. Osada . 2021. “Life Cycle Assessment of Sustainable Broiler Production Systems: Effects of Low‐Protein Diet and Litter Incineration.” Agriculture 11: 921. 10.3390/agriculture11100921.

[asj70129-bib-0036] Ogino, A. , T. Osada , R. Takada , et al. 2013. “Life Cycle Assessment of Japanese Pig Farming Using Low‐Protein Diet Supplemented With Amino Acids.” Soil Science & Plant Nutrition 59: 107–118. 10.1080/00380768.2012.730476.

[asj70129-bib-0037] Pelletier, N. 2017. “Life Cycle Assessment of Canadian Egg Products, With Differentiation by Hen Housing System Type.” Journal of Cleaner Production 152: 167–180. 10.1016/j.jclepro.2017.03.050.

[asj70129-bib-0038] Rondoni, A. , D. Asioli , and E. Millan . 2020. “Consumer Behaviour, Perceptions, and Preferences Towards Eggs: A Review of the Literature and Discussion of Industry Implications.” Trends in Food Science and Technology 106: 391–401. 10.1016/j.tifs.2020.10.038.

[asj70129-bib-0039] Sakomura, N. K. 2004. “Modeling Energy Utilization in Broiler Breeders, Laying Hens and Broilers.” Brazilian Journal of Poultry Science 6: 1–11. 10.1590/S1516-635X2004000100001.

[asj70129-bib-0040] Sakomura, N. K. , R. Silva , H. P. Couto , C. Coon , and C. R. Pacheco . 2003. “Modeling Metabolizable Energy Utilization in Broiler Breeder Pullets.” Poultry Science 82: 419–427. 10.1093/ps/82.3.419. 12705403

[asj70129-bib-0041] Setoguchi, A. , K. Oishi , Y. Kimura , A. Ogino , H. Kumagai , and H. Hirooka . 2022. “Carbon Footprint Assessment of a Whole Dairy Farming System With a Biogas Plant and the Use of Solid Fraction of Digestate as a Recycled Bedding Material.” Resources, Conservation & Recycling Advances 15: 200115. 10.1016/j.rcradv.2022.200115.

[asj70129-bib-0042] Summers, J. D. 1993. “Reducing Nitrogen Excretion of the Laying Hen by Feeding Lower Crude Protein Diets.” Poultry Science 72: 1473–1478. 10.3382/ps.0721473. 8378220

[asj70129-bib-0043] Swelum, A. A. , M. T. El‐Saadony , M. E. Abd El‐Hack , et al. 2021. “Ammonia Emissions in Poultry Houses and Microbial Nitrification as a Promising Reduction Strategy.” Science of the Total Environment 781: 146978. 10.1016/j.scitotenv.2021.146978.

[asj70129-bib-0044] Weeks, C. A. , S. L. Lambton , and A. G. Williams . 2016. “Implications for Welfare, Productivity and Sustainability of the Variation in Reported Levels of Mortality for Laying Hen Flocks Kept in Different Housing Systems: A Meta‐Analysis of Ten Studies.” PLoS ONE 11: e0146394. 10.1371/journal.pone.0146394. 26734933 PMC4703395

